# Short-term and long-term outcomes in patients with cryptococcal meningitis after ventriculoperitoneal shunt placement

**DOI:** 10.3389/fneur.2022.773334

**Published:** 2022-11-17

**Authors:** Junxian Wen, Rui Yin, Jianbo Chang, Yihao Chen, Xiying Dong, Wei Cao, Xiaojun Ma, Taisheng Li, Junji Wei

**Affiliations:** ^1^Department of Neurosurgery, Peking Union Medical College Hospital, Peking Union Medical College, Chinese Academy of Medical Sciences, Beijing, China; ^2^Department of Infectious Disease, Peking Union Medical College Hospital, Peking Union Medical College, Chinese Academy of Medical Sciences, Beijing, China

**Keywords:** cryptococcal meningitis, short-term outcomes, long-term outcomes, ventriculoperitoneal shunt, survival analysis

## Abstract

**Objective:**

The purpose of this study was to assess the short-term and long-term outcomes of ventriculoperitoneal shunt (VPS) placement in patients with cryptococcal meningitis (CM).

**Methods:**

We performed a retrospective analysis of all patients with CM admitted to the Peking Union Medical College Hospital from September 1990 to January 2021. We collected related clinical features to analyze the short- and long-term outcomes of VPS at 1 month and 1 year at least the following therapy, respectively. Overall survival (OS) was compared with all patients and a subgroup of critically ill cases by the Kaplan–Meier method with the log-rank test. Univariable and multivariable analyses were also performed using the Cox proportional hazard model to identify statistically significant prognostic factors.

**Results:**

We enrolled 98 patients, fifteen of whom underwent VPS. Those who received VPS had a lower cerebrospinal fluid (CSF) Cryptococcus burden (1:1 vs. 1:16; *p* = 0.046), lower opening pressures (173.3 mmH_2_ O vs. 224 mmH_2_O; *p* = 0.009) at lumbar punctures, and a lower incidence of critical cases (6.7 vs. 31.3%; *p* = 0.049). According to our long-term follow-up, no significant difference was shown in the Barthel Index (BI) between the two groups. Two patients in the VPS group suffered postoperative complications and had to go through another revision surgery. According to survival analysis, overall survival (OS) between the VPS and non-VPS groups was not significantly different. However, the Kaplan–Meier plots showed that critical patients with VPS had better survival in OS (*p* < 0.009). Multivariable analyses for critical patients showed VPS was an independent prognostic factor.

**Conclusion:**

A VPS could reduce the intracranial pressure (ICP), decrease the counts of *Cryptococcus neoformans* by a faster rate and reduce the number of critical cases. The VPS used in critical patients with CM has a significant impact on survival, but it showed no improvement in the long-term Barthel Index (BI) vs. the conservative treatment and could lead to postoperative complications.

## Introduction

Cryptococcosis is a fungal disease with high mortality, which presents as a major opportunistic infection of patients with impaired cellular immunity, especially for those with AIDS and diabetes (1–3). Cryptococcosis is acquired *via* inhalation of the spores of *Cryptococcus neoformans*, a yeast-like round or oval fungus that is found primarily in the soil and is widely distributed across the globe (2). Cryptococcosis commonly involves the respiratory and central nervous systems (3). Cryptococcal meningitis (CM) is the most serious condition in the spectrum of diseases caused by *C. neoformans*, which is also the most common life-threatening fungal infection. Complications of CM include fever, headache, neck stiffness, visual impairment, cranial nerve palsy, ataxia, and epilepsy (3–5). Furthermore, misdiagnosis and diagnostic delay of CM are associated with a worse neurological outcome and higher mortality (6). Therefore, it is essential to recognize the cause of diagnostic errors to improve clinical outcomes.

Treatment for cryptococcal meningitis is currently an antifungal therapy comprised of amphotericin B (AmB) plus 5-flucytosine (5-FC) or fluconazole (3, 5). However, persistent and uncontrollable intracranial hypertension and difficulty in reducing the Cryptococcus count pose a severe threat to patients with CM. If elevated CSF pressure is not properly managed, it can lead to the severe neurological deficit and even death (7). The ventriculoperitoneal shunt (VPS) has been studied extensively with respect to its efficacy in patients with high intracranial pressure (ICP). Its validity is certain and has been generally accepted, which is in accordance with the clinical practices in our center—a VPS would be recommended if (1) refractory elevated ICP >35 cmH_2_O, (2) the effect of conservative treatment is limited, or (3) severe clinical manifestations, such as damage to cranial nerve function. Current guidelines also recommend that if the patient is receiving or has received appropriate antifungal therapy, VPS could be placed. Furthermore, if necessary, a VPS can be placed during active infection without complete sterilization of CSF (8, 9). Some articles have reported the efficiency of permanent shunt placement in patients with cryptococcal meningitis (10–12). However, it lacked data on the long-term follow-ups of patients after surgeries.

Here, we present a single-center study of patients with cryptococcal meningitis, with a comprehensive analysis of clinical features and outcomes. We aim to analyze the short- and long-term efficacies of VPS placement in patients with CM.

## Materials and methods

### Patient selection and enrollment

A retrospective electronic chart review was conducted on the medical records from Peking Union Medical College Hospital. Based on the International Classification of Diseases, 9^th^ Revision (ICD-9), patients with admission and discharge diagnoses of cryptococcal meningitis admitted between September 1990 and January 2021 were identified. Patients were required to meet all of the following criteria for the diagnosis of cryptococcal meningitis (3): (1) *C. neoforman* was detected in CSF samples; (2) positive titer for CSF cryptococcal antigen; (3) clinical manifestations of CM with *C. neoformans* detected in blood cultures or a CSF smear tested positive for India ink stain. The exclusion criteria were as follows: (1) evidence of meningitis due to other pathogens; and (2) lack of follow-up data.

These selected patients with CM were divided into two groups based on whether they had a VPS placement. All recruited patients were given standard antifungal therapy. We collected all patients' demographic data, underlying diseases, clinical presentation, diagnostic laboratory and radiologic findings, and clinical outcomes. The baseline clinical features of the two groups were compared. Critical status was defined as: (1) when a patient had been admitted to the intensive care unit or the emergency department, or a patient was in a palliative condition; (2) patients with uncontrollable intracranial hypertension; and (3) patients with rapid vision loss, hearing impairment, or other cranial nerve palsies. The diagnosis of the critical status of patients with CM was made by two experienced physicians in our hospital based on the patient's characteristics and clinical data. If the opinions are inconsistent, they will be resolved through group discussion. The short-term outcome of the treatment response was assessed 1 month after the initiation of antifungal therapy for the non-VPS group and 1 month after surgery for the VP group, respectively. At least 1 year following therapy, patients were interviewed by means of phone calls from a trained interviewer to assess their long-term outcomes. Interviews took an average of 10 min. The questionnaire included detailed questions about patients' physical condition, current clinical symptoms and signs, postoperative complications if they have had VPS placement, and activity of daily living (ADL). The Barthel Index (BI) was used to assess the ADL by 10 items (grooming, bathing, toilet use, bowel movement, urinary incontinence, dressing, feeding, stair climbing, transferring, and walking) (13).

Informed consent was obtained from all patients upon hospitalization. This retrospective study was performed under the authorization of the institutional ethics committee of PUMCH, Chinese Academy of Medical Sciences.

### Data analysis

**Figure 1:**
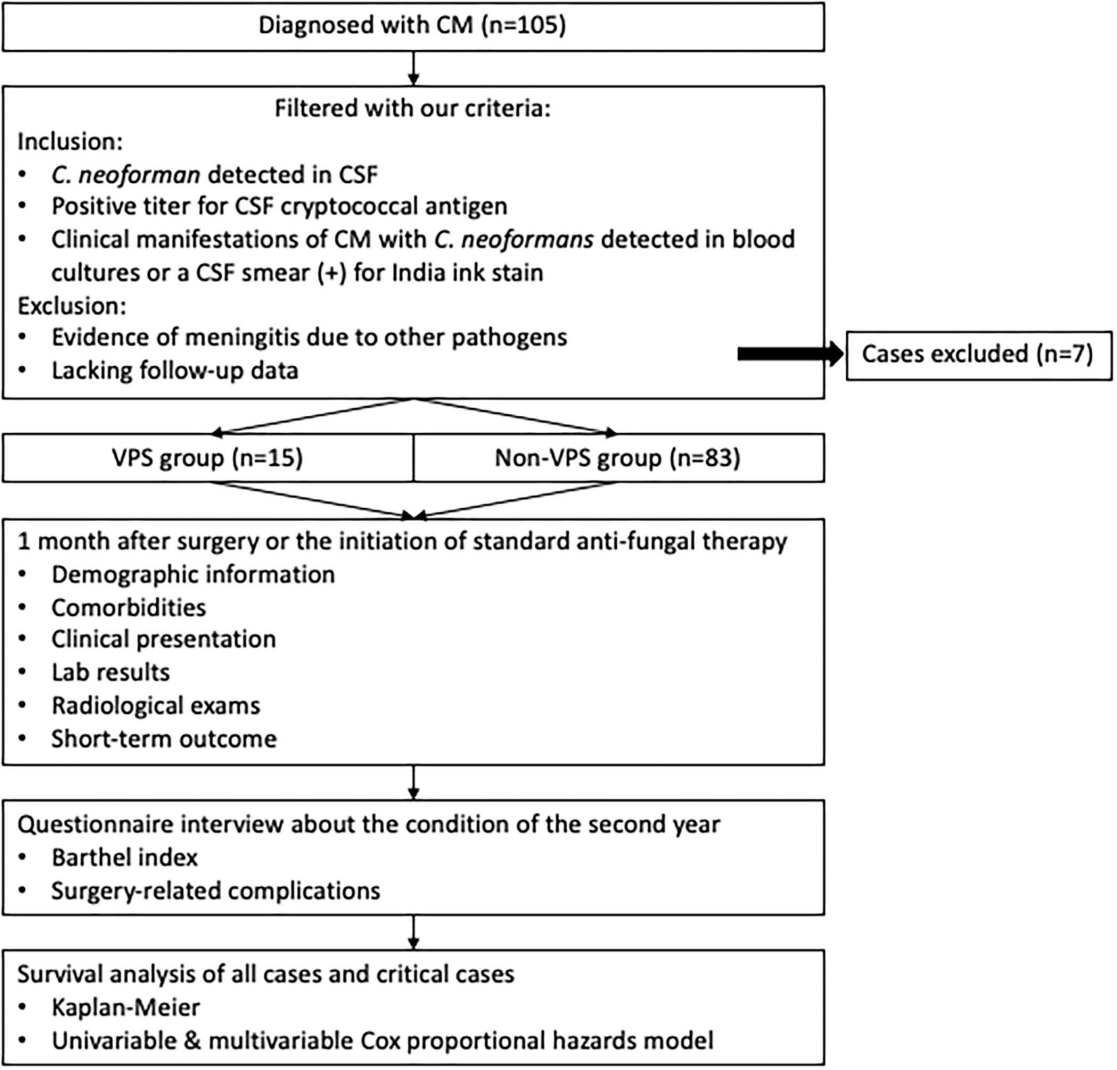
Study flow diagram.

All data were analyzed using SPSS software version 26.0 (IBM Corp., Armonk, New York, USA). Continuous variables are reported as the mean ± standard deviation (SD) or median [interquartile range (IQR)]; numbers and percentages are used for categorical variables. We compared baseline characteristics and the short- and long-term outcomes between the VPS group and the non-VPS group. Univariate analyses between the two groups were performed using the chi-square test and Fisher's exact test for categorical variables. The Wilcoxon–Mann–Whitney test was employed for continuous variables that did not fit a normal distribution. The *p*-values of 0.05 (two-tailed) or less were considered statistically significant. Survival was estimated using the Kaplan–Meier method and compared between the VPS and non-VPS groups using a log-rank test. Hazard ratios (*HR*s) and 95% confidence intervals (*CI*s) were calculated by the univariate and multivariate Cox proportional hazard models to assess the relative contribution of factors to the survival of CM. Moreover, in order to figure out the effect of VPS on the OS of critical patients, we also performed a survival analysis specifically for this subgroup. The study flow is presented in Figure 1.

## Results

### Demographics

During the study period, 105 cases were included, and seven cases were excluded according to the criteria above. Finally, 98 cases were enrolled. Depending on whether the VPS was placed, the cases were divided into a VPS group (*n* = 15) and a non-VPS group (*n* = 83). The baseline clinical features of the two groups were documented and presented in the baseline characteristics of patients with cryptococcal meningitis (Table 1). The median age of the cohort was 37.5 (29.0–50.7) years, 56.1% were men, and 25% of people had confirmed contact with birds. The median time between diagnosis and surgery in the VPS group was 88.5 (64.75–100) days. The most common underlying conditions were a history of corticosteroid use (40.8%) and rheumatism diseases (32.7%). No significant differences were observed between the VPS and non-VPS groups regarding these variables.

**Table 1:** Clinical characteristics of patients with CM.

**Variable**	**Total**	**VPS**	**Non-VPS**	***P*** **-value**
	***N*** **= 98 (%)**	***N*** **= 15 (%)**	***N*** **= 83 (%)**	
**Age (range)**	37.5 (29.0–50.7)	47.0 (32.5–50.5)	46.0 (28.5–50.5)	0.351
**Sex**				0.813
Male	55 (56.1)	8 (53.3)	4*7 (*46.7)	
Female	43 (43.9)	7 (46.7)	36 (43.4)	
**Bird contact history**	25 (25.5)	4 (26.7)	21 (25.3)	0.911
**Time between diagnosis and surgery (range)**		88.5 (64.75–100)		
**Misdiagnosis**	37 (37.8)	7 (46.7)	30 (36.1)	0.439
Tuberculous meningitis	15 (15.3)	3 (20.0)	12 (14.5)	0.513
Viral meningitis	3 (3.1)	1 (6.7)	2 (2.4)	0.378
Other meningitis	4 (4.1)	0 (0.0)	4 (4.8)	0.385
SLEE	4 (4.1)	1 (6.7)	3 (3.6)	0.582
Upper respiratory infection	2 (2.0)	0 (0.0)	2 (2.4)	0.544
Other diseases	9 (9.2)	2 (13.3)	7 (8.4)	0.545
**Clinical presentation**				
Headache	88 (89.8)	14 (93.3)	74 (89.2)	0.623
Fever	85 (86.7)	11 (73.3)	74 (89.2)	0.096
Vomiting	60 (61.2)	15 (100)	45 (50.8)	**0.001**
Visual impairment	34 (34.7)	9 (60.0)	25 (30.1)	**0.025**
Altered mental status	32 (33.0)	7 (46.7)	25 (30.5)	0.220
Meningeal irritation	34 (34.7)	10 (66.7)	24 (28.9)	**0.005**
Papilledema	27 (27.6)	8 (53.3)	19 (22.9)	**0.015**
Cranial nerve palsy	24 (24.5)	7 (46.7)	17 (20.5)	**0.030**
Hearing impairment	11 (11.2)	2 (13.3)	9 (10.8)	0.779
Seizures	11 (11.2)	2 (13.3)	9 (10.8)	0.779
**Underlying disease**				
Corticosteroid use	40 (40.8)	5 (33.3)	35 (42.2)	0.522
Rheumatological disease	32 (32.7)	4 (26.7)	28 (33.7)	0.591
Hepatitis	9 (9.2)	1 (6.7)	8 (9.6)	0.714
Tuberculosis	8 (8.2)	1 (6.7)	7 (8.4)	0.818
HIV	5 (5.1)	0 (0.0)	5 (6.0)	0.329
Cancer	2 (2.1)	0 (0.0)	2 (2.4)	0.541
**Brain images (CT or MRI**, ***n*** **=** **87)**				
Hydrocephalus	39 (40.2)	13 (86.7)	26 (31.7)	**<0.001**
Cryptococcosis-related suspicious lesions	21 (21.4)	2 (13.3)	19 (22.9)	0.406
Meningeal enhancement	14 (14.3)	3 (20.0)	11 (13.3)	0.492
Cerebral edema	7 (7.1)	3 (20.0)	4 (4.8)	**0.036**
**CSF testing**				
Positive CSF culture	60 (53.1)	12 (80.0)	46 (55.4)	0.075
India ink staining	69 (71.9)	13 (86.7)	56 (69.1)	0.165
Opening pressure mmH_2_0 (range)	275 (200–330)	330 (325–330)	260 (197.5–310)	**0.002**
WBC count × 10^6^/l (range)	24 (6–83)	30 (16–76)	33.5 (8.75–93.5)	0.878
Protein g/l (range)	1.05 (0.61–1.76)	1.21 (0.76–1.76)	1.03 (0.59–1.74)	0.383
Glucose mmol/l (range)	2.00 (1.16–2.80)	2.00 (1.25–3.45)	1.95 (1.17–2.71)	0.475
Cryptococcal antigen (range)	512 (8–512)	384 (48–1,024)	512 (6–512)	0.741

SLEE, systemic lupus erythematosus encephalopathy; HIV, human immunodeficiency virus; CT, computerized tomography; MRI, magnetic resonance imaging; CSF, cerebrospinal fluid; WBC, white blood cell.

The most common clinical feature was headache (80.5%), followed by fever (78.8%) and vomiting (54.9%). The symptoms or signs most strongly associated with VPS were vomiting (100 vs. 50.8%; *p* = 0.001), meningeal irritation (66.7 vs. 28.9%; *p* = 0.005), papilledema (53.3 vs. 22.9; *p* = 0.015), and cranial nerve palsy (46.4 vs. 20.5%; *p* = 0.030). The remaining symptoms were similar among the two groups.

Hydrocephalus was present in 40.2% of all enrolled patients and its incidence was greater among the VPS group (86.7 vs. 31.7%; *p* < 0.001). In addition, the incidence of cerebral edema was greater (20.0 vs. 4.8%; *p* < 0.001). The median opening pressure measured by lumbar punctures was 275 mmH_2_O, and the subgroup level was greater in patients who were shunted (330 vs. 260 mmH_2_O; *p* = 0.002).

### Short-term outcomes

At 1 month after the surgery in the patients with VPS and 1 month after antifungal therapy in the non-VPS cases, important clinical factors were recorded to assess short-term prognoses, respectively. There was no significant difference in medication regimen between the two groups of patients for the same period. The characteristics of these participants are shown in Table 2. Most patients had a certain degree of improvement in their symptoms and signs following 1 month of surgery or antifungal treatment. However, no significant difference was observed between the two groups (Table 2). Lumbar punctures were performed in all patients. Those patients who received VPS had lower CSF cryptococcus burden (1:1 vs. 1:16; *p* = 0.046) and lower opening pressures (170 vs. 220 mmH_2_O; *p* = 0.009) vs. the non-VPS group. Other than those, there were no significant differences in either the incidences of positive CSF culture and positive India ink stain, or the counts of white blood cells (WBC), neutrophils, protein, and glucose in CSF. Hospital mortalities were similar between the two groups; however, the incidence of critical cases was greater among patients who were not shunted (6.7 vs. 31.3%; *p* = 0.049).

**Table 2:** The short-term outcomes of VPS and non-VPS patients.

**Variable**	**VPS**	**Non-VPS**	***P*** **-value**
	***N*** **= 15 (%)**	***N*** **= 83 (%)**	
**Symptom and sign improved**			
Altered mental status	4/7	15/22	0.665^*^
Headache	10/14	51/67	0.711
Fever	7/11	53/67	0.259
Vomiting	14/15	39/42	1.000^*^
Papilledema	6/8	13/15	0.589^*^
Visual impairment	8/9	21/22	0.503
Hearing impairment	1/2	2/6	1.000^*^
Cranial nerve palsy	7/7	7/8	1.000^*^
**CSF testing**			
Positive CSF culture	2 (13.1)	17 (20.5)	0.519
India ink staining	4 (26.7)	35 (43.2)	0.231
Opening pressure mmH_2_O (range)	170 (140–190)	220 (165–275)	**0.009**
WBC count × 10^6^/l (range)	4 (3–23.5)	6 (0–37.5)	0.889
Protein g/l (range)	0.94 (0.60–1.25)	0.61 (0.38–1.145)	0.113
Glucose mmol/l (range)	2.60 (2.40–3.15)	2.70 (2.20–3.20)	0.906
Cryptococcus count (range)	1 (0–64)	16 (1–256)	**0.046**
**Adverse outcome**			
Critical cases	1 (6.7)	26 (31.3)	**0.049**
Death	0 (0.0)	2 (2.4)	1.000^*^

CSF, cerebrospinal fluid; WBC, white blood cell.

* Fisher exact test.

### Questionnaire-based long-term clinical survey

In total, 13 VPS and 58 non-VPS cases completed the survey and were included in the analysis for long-term outcomes. The follow-up rate was 72.4% with 27 patients lost to follow-up. The average duration of follow-ups was for 6.8 years. All patients' ability to complete activities of daily living was measured according to the Barthel Index: (a) feeding, (b) bathing, (c) grooming, (d) dressing, (e) bowels program, (f) bladder program, (g) toilet use, (h) bed-wheelchair transfers, (i) mobility, and (j) stair-climbing. However, as listed in Table 3, there was no significant difference in the final BIs between the two groups. Moreover, two patients with VPS suffered postoperative complications (one with an infection at the site of an abdominal incision and one with a broken tube), and both underwent revision surgeries.

**Table 3:** Follow up with VPS and non-VPS patients at least 1 years after they were discharged from hospital.

**Variable**	**VPS**	**Non-VPS**	***P*** **-value**
	**(*N* = 13)**	**(*N* = 58)**	
**Barthel index (SD)**			
Feeding	10 (0)	9.0 (2.4)	0.497
Bathing	5.5 (1.5)	4.0 (2.0)	0.214
Grooming	5 (1.5)	4.0 (1.9)	0.214
Dressing	10 (0)	8.8 (2.9)	0.497
Bowels	10 (0)	9.6 (1.9)	0.876
Bladder	9 (3.1)	9.6 (1.9)	0.794
Toilet use	10 (0)	9.2 (2.7)	0.741
Bed-wheelchair transfers	14.5 (1.5)	13.2 (4.2)	0.664
Mobility	14 (2.1)	12.6 (4.7)	0.794
Stairs	10 (7.6)	7.1 (3.7)	0.256
Total	97.5 (5.4)	84.2 (29.2)	0.242
**Postoperative complications**	2	–	**–**
Infection	1		
Breakage of the tube	1		

SD, standard deviation.

### Survival analysis

Of all the 71 patients finally recruited, 34 patients had died by the end of the last follow-up. The Kaplan–Meier survival analysis shows that the overall survival (OS) of the two groups was not statistically significant different (*p* = 0.103). In order to further figure out the effect of VPS on the OS of patients with CM, we applied the Cox proportional hazard model to the analysis. However, the result was consistent with the Kaplan–Meier analysis. Patients with VPS had a similar hazard ratio (HR) of 2.543 [95% confidence interval (CI) = 0.777–8.322, *p* = 0.123] when compared with the non-VPS group.

**Figure 2:**
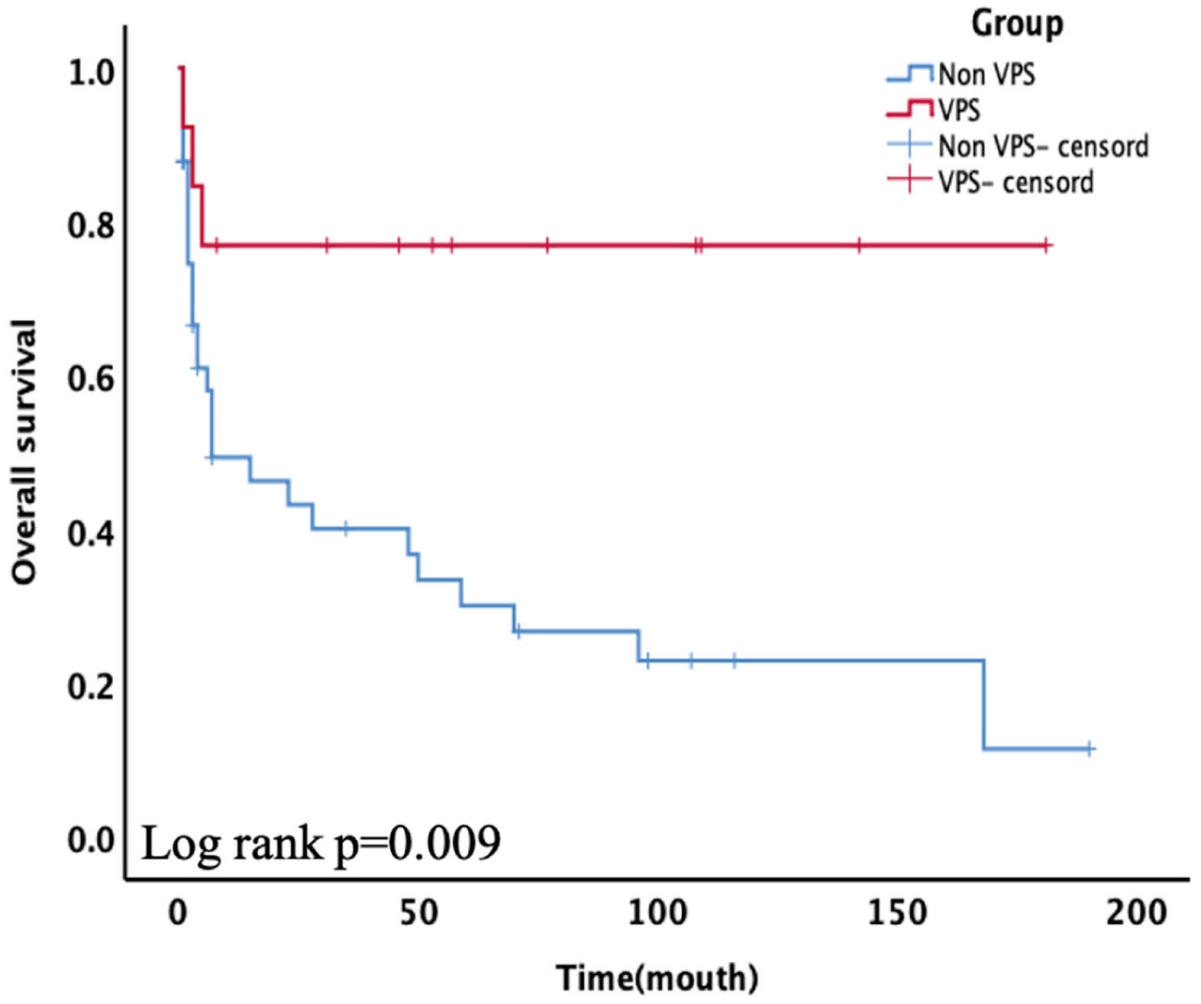
Subgroups of critical patients receiving ventriculoperitoneal shunt (VPS) presented significantly higher overall survival (OS) than the non-VPS group (*p* = 0.009).

Nonetheless, a significant difference was observed between the critical case groups. The Kaplan–Meier plot of survival time is shown in Figure 2. Among critically ill patients, patients with VPS had a better mean survival time than non-VPS (139.92 ± 20.80 vs. 55.94 ± 12.39, *p* = 0.009). In the univariable analysis and multivariate cox analysis for critical patients (Table 4), VPS still showed statistical differences (HR 8.432, 95% CI 0.983–72.310, *p* = 0.020).

**Table 4:** Cox proportional hazards analysis for critical patients with cryptococcal meningitis.

**Variable**	**Univariate regression**			**Multivariate regression**		
	***P-*** **value**	**Hazard ratio**	**95%CI**	***P*** **-value**	**Hazard ratio**	**95%CI**
Underlying disease: cancer	0.032	10.4	1.217–89.036			
VP	0.018	4.196	1.272–13.837	0.020	8.432	0.983–72.310

CI, confidence interval.

## Discussion

Cryptococcal meningitis is one of the most common fungal infections in immunodeficient patients, especially in those with HIV (14). Meanwhile, the increasing number of organ transplantations and the widespread use of immunosuppressive therapies have led to a dramatic increase in the incidence of non-HIV-associated cryptococcosis (15, 16). In our study, we also found that corticosteroid use and rheumatological and liver diseases were some of the other major underlying diseases. Published data have shown that there are about 223,100 estimated cases of CM, and the overall mortality from CM in resource-limited settings is ~70% (17, 18). An elevated CSF pressure has been proven to be correlated with increased mortality and worse outcomes (19, 20). Many studies have proved that VPS or external ventricular drainage is an effective method in relieving high intracranial pressure of patients with CM with or without ventriculomegaly (16, 21). Some authors suggest prophylactic shunt placement avoid irreversible neurological complications (22, 23). However, for some patients with CM, diversion of CSF to the peritoneal cavity through a VPS could lead to various postoperative complications (9). Additionally, the duration of follow-ups in previous studies was short (12, 21, 23). Thus, here we were the first to conduct a long-term follow-up of patients with CM that lasted more than a year to analyze the long-term efficacy and safety of VPS in patients with CM.

In our study, one-third of patients were misdiagnosed at the initial visit. The reason may be that most of our patients were identified with non-HIV associated CM. They had other underlying conditions, such as rheumatological diseases, a history of corticosteroid use, and cancer. Unlike HIV-associated CM, immunocompetent patients did not have a high cryptococcus load. Thus, cultures and antigen tests of their CSF may sometimes turn out negative, and the diagnosis is hard to exclude (14). Additionally, as listed in Table 1, there were several clinical presentations and laboratory or radiographic parameters that were independently associated with the likelihood of receiving a subsequent VPS. Initially, patients with CM often present with headaches, fever, vomiting, and altered mental status over several weeks. As the disease progresses, the viscosity of CSF is elevated and a large number of *C. neoformans* and inflammatory cell infiltrates may block the channels and valves of the subarachnoid villi, thus occluding the normal circuit of CSF flow and elevating the intracranial pressure (24–26). There is an increase in the opening pressure of the lumbar tap, and related symptoms could also exacerbate. CT and MRI scans may present with meningeal enhancement, single or multiple nodules, cerebral edema, or hydrocephalus (14). Doctors may prefer to surgically treat patients whose imagining results showed these characteristics.

As suggested by the Infectious Disease Society of America (IDSA) 2010 guidelines, CM management still relies on the three-phase antifungal approach of induction, consolidation, and maintenance (8). The common regimen is AmB (0.7–1.0 mg/kg per day) plus 5-FC (100 mg/kg per day) for at least 4 weeks as the induction therapy, followed by consolidation with fluconazole for 8 weeks (8, 27). In addition, increased ICP in patients with CM is associated with short-term death and neurological deficiency. If there is a persistent ICP elevation, permanent VPS placement is recommended in many studies (8, 23). In short-term outcomes, we found that the VPS could effectively reduce ICP, lower the Cryptococcus antigen count in a faster manner and reduce the number of critical cases, which agrees with the earlier reports (10, 21, 23). With VPS placed, the obstruction to CSF flow would be once-and-for-all solved. Moreover, the VPS with adjustable valve reservoirs had advantages in adjusting the amount of CSF drainage and assessing the shunt's patency (28). It was also observed that VPS can significantly reduce the cryptococcus count in the CSF in our study (*p* = 0,046). The reasons could be: (1) With VPS placed, *C. neoformans* are drained into the peritoneal cavity. Compared with CSF, there is a dramatic decrease in the amount of inositol in the peritoneal fluid. However, inositol is essential for cryptococcus to complete its sexual cycle and regulate intracellular signaling (29). (2) In addition, some studies found that *C. neoformans* can directly attach to the endothelial surface of the brain microvasculature and secret hyaluronic acid, urease, phospholipase B, and the extracellular protease Mpr1 to promote the process of binding to endothelial cells, which lead to the *C. neoformans* crossing the blood-brain barrier (20, 30–32), but similar mechanisms did not show the same result in the blood–peritoneal barrier. Thus, as the burden of cryptococcus was brought down and the ICP dropped, the incidence of critical cases also decreased.

Currently, there were no studies with a long-term follow-up period of more than 1 year. Our study is the first to include patients with CM in such a timeframe. Two patients suffered postoperative complications during the follow-up period. Both infection and shunt obstruction are common complications associated with VPS. The spectrum and proportion of postoperative complications were not different from those reported in similar studies (9, 12, 23, 33). Stringent adherence to the aseptic technique, proper placement, and fixation of the shunt may reduce these risks. But once the postoperative infection or tube obstruction occurs, the revision surgery should be considered (34). Fortunately, salvage surgery had no significant influence on the long-term BIs of these two patients. They both achieved full scores. As shown in the survival analysis, the VPS was associated with significantly improved OS in critical cases but not all patients. We hypothesized that VPS placement could decrease the CSF pressure, *C. neoformans* counts more quickly, and thus the life-threatening risk of CM patients could be reduced (22). Furthermore, after surgery, the ICPs of the VPS group patients were closely monitored and exquisitely regulated so that any emergent incident can be dealt with in time. We have considered that VPS is an effective means to cross the risk period for critical patients with CM. However, there were no significant differences found in any items of the BI between the two groups. The possible reason could be when patients come to a maintenance stage, the advantages brought about by VPS would become modest. Therefore, from our long-term follow-up results and survival analysis, we concluded that early VPS should be applied to avoid death for patients with CM with uncontrollable intracranial hypertension and rapid development of clinical symptoms. Once a patient has gone through the risk period, surgical treatment cannot improve the long-term BI of patients with CM. Thus, indications for VPS placement in patients with CM should be carefully evaluated.

There are some limitations to this study. First, the study design was retrospective, which limits the evaluation of causality. Moreover, the collection of long-term follow-up data with only a tele-questionnaire is not comprehensive enough. Other limitations of this study were the small number of cases and the unbalanced size of the groups examined. Furthermore, changes in the therapies of CM and standard operation procedures of VPS placement may lead to difficulties in interpreting the results. Therefore, large-scale prospective studies are needed in the future.

## Conclusion

In summary, VPS could effectively relieve high ICP, have a more rapid clearance of *C. neoformans* counts, and reduce the incidence of critical cases in patients with CM. Early VPS used in critical patients with CM has a significant impact on survival, but it cannot improve the long-term BI and could bring out operative complications. Indications for placing permanent VPS in patients with CM should be carefully evaluated.

## Data availability statement

The original contributions presented in the study are included in the article/supplementary material, further inquiries can be directed to the corresponding author.

## Author contributions

JXW did the main study analysis and co-wrote the manuscript. RY did the analysis of clinical data and co-wrote the manuscript. XYD, YHC, and JBC collected the medical records and managed data processes. WC and XJM participated in making the antifugal strategy and reviewed the results. TSL reviewed the study results and edited the manuscript. JJW edited the manuscript. All authors revised and approved the manuscript.

## Funding

This research received grants from (1) National Key R&D Program of China (2018YFA0108603); (2) Beijing Tianjin Hebei basic research cooperation project [19JCZDJC64600(Z)]; (3) National High Level Hospital Clinical Research Funding (2022-PUMCH-032). These projects support the design of the study and collection, analysis, and interpretation of data.

## Conflict of interest

The authors declare that the research was conducted in the absence of any commercial or financial relationships that could be construed as a potential conflict of interest.

## Publisher's note

All claims expressed in this article are solely those of the authors and do not necessarily represent those of their affiliated organizations, or those of the publisher, the editors and the reviewers. Any product that may be evaluated in this article, or claim that may be made by its manufacturer, is not guaranteed or endorsed by the publisher.
